# Association between genetic variant on chromosome 12p13 and stroke survival and recurrence: a one year prospective study in Taiwan

**DOI:** 10.1186/1423-0127-19-1

**Published:** 2012-01-03

**Authors:** Yi-Chen Hsieh, Sudha Seshadri, Wen-Ting Chung, Fang-I Hsieh, Yi-Hsiang Hsu, Huey-Juan Lin, Hung-Pin Tseng, Li-Ming Lien, Chyi-Huey Bai, Chaur-Jong Hu, Jiann-Shing Jeng, Sung-Chun Tang, Chin-I Chen, Chia-Chen Yu, Hung-Yi Chiou

**Affiliations:** 1 School of Public Health, Taipei Medical University, Taipei, Taiwan; 2 Department of Neurology, Boston University School of Medicine, Framingham Heart Study, Boston, MA, USA; 3 National Heart, Lung, and Blood Institute's Framingham Heart Study, Framingham, MA, USA; 4 Department of Neurology, Wanfang Hospital, Taipei Medical University, Taipei, Taiwan; 5 Graduate Institute of Clinical Medicine, Taipei Medical University, Taipei, Taiwan; 6 Dr. Chi-Hsing Huang Stroke Research Center, Taipei Medical University, Taipei, Taiwan; 7 Institute for Aging Research, Hebrew SeniorLife and Harvard Medical School, Boston, MA 02131, USA; 8 Molecular and Integrative Physiological Science Program, Harvard School of Public Health; 9 Department of Neurology, Chi-Mei Medical Center, Tainan, Taiwan; 10 Department of Neurology, Lotung Poh-Ai Hospital, I-Lan, Taiwan; 11 Department of Neurology, Shin Kong Wu Ho-Su Memorial Hospital, Taipei, Taiwan; 12 Central Laboratory, Shin Kong Wu Ho-Su Memorial Hospital, Taipei, Taiwan; 13 Department of Neurology, Taipei Medical University Hospital and Shuang Ho Hospital, Taipei, Taiwan; 14 Stroke Center and Department of Neurology, National Taiwan University Hospital, Taipei, Taiwan

**Keywords:** stroke, single nucleotide polymorphisms, survival

## Abstract

**Background:**

The association between ischemic stroke and 2 single nucleotide polymorphisms (SNPs) on chromosome 12p13, rs12425791 and rs11833579 appears inconsistent across different samples. These SNPs are close to the ninjurin2 gene which may alter the risk of stroke by affecting brain response to ischemic injury. The purpose of this study was to investigate the association between these two SNPs and ischemic stroke risk, as well as prognostic outcomes in a Taiwanese sample.

**Methods:**

We examined the relations of these two SNPs to the odds of new-onset ischemic stroke, ischemic stroke subtypes, and to the one year risk of stroke-related death or recurrent stroke following initial stroke in a case-control study. A total of 765 consecutive patients who had first-ever ischemic stroke were compared to 977 stroke-free, age-matched controls. SNPs were genotyped by Taqman fluorescent allelic discrimination assay. The association between ischemic stroke and SNPs were analyzed by multivariate logistic regression. Cox proportional hazard model was used to assess the effect of individual SNPs on stroke-related mortality or recurrent stroke.

**Results:**

There was no significant association between SNP rs12425791 and rs11833579 and ischemic stroke after multiple testing corrections. However, the marginal significant association was observed between SNP rs12425791 and large artery atherosclerosis under recessive model (OR, 2.30; 95%CI, 1.22-4.34; q-value = 0.062). Among the 765 ischemic stroke patients, 59 died or developed a recurrent stroke. After adjustment for age, sex, vascular risk factors and baseline stroke severity, Cox proportional hazard analysis indicated that the hazard ratios were 2.76 (95%CI, 1.34-5.68; q-value, 0.02) and 2.15 (95%CI, 1.15-4.02; q-value, 0.03) for individuals with homozygous variant allele of rs12425791 and rs11833579, respectively.

**Conclusions:**

This is a precedent study that found genetic variants of rs12425791 and rs11833579 on chromosome 12p13 are independent predictors of stroke-related mortality or stroke recurrence in patients with incident ischemic stroke in Taiwan. Further study is needed to explore the details of the physiological function and the molecular mechanisms underlying the association of this genetic locus with ischemic stroke.

## Background

A genome-wide association study (GWAS) conducted by the Cohorts for Heart and Aging Research Consortium in Genetic Epidemiology (CHARGE) reported a novel association between ischemic stroke and 2 single nucleotide polymorphisms (SNPs) on chromosome 12p13, rs12425791 and rs11833579, in a Caucasian sample [1]. The researchers further examined the association between the two implicated SNPs and ischemic stroke in two independent replication samples, an African-American community-based cohort and a Dutch case-control. From both samples, they observed that rs12425791 was significantly associated with the incidence of ischemic stroke, particular in atherothrombotic stroke [1]. Nevertheless, the results of subsequent validated studies have been inconsistent. The International Stroke Genetics Consortium (ISGC) and the Wellcome Trust Case-Control Consortium 2 (WTCCC2) performed a meta-analysis combining case-control data among European ancestry and a population-based genomewide cohort study; however, the results were controversial [2]. In addition, an Italian and a Swedish case-control study also failed to show any associations between the two genetic variants on chromosome 12p13 and ischemic stroke [3,4]. Conversely, a large population-based case-control study found a significant association between atherothrombotic stroke and rs12425791 in Japan [5].

The 2 SNPs on chromosome 12p13, rs12425791 and rs11833579 are in close proximity to the gene *NINJ2*, which encodes ninjurin2, a member of the 'ninjurin' or nerve-injury-induced protein family [6]. *NINJ2 *plays a role in nerve regeneration and may increase the risk of stroke by altering brain response to ischemic injury. If this were true, the minor allele that increases stroke risk would also be associated with greater stroke severity and disability. In this case-control study, we investigated the association between the two SNPs, rs12425791 and rs11833579 and the risk of ischemic stroke among Taiwanese population who had first-ever ischemic strokes. Furthermore, the study also examined whether the associations between these two SNPs and end-point of stroke-related mortality or recurrent stroke were present.

## Methods

### Subject recruitment and study design

The study was conducted in the Formosa Stroke Genetic Consortium (FSGC), a platform for hospital collaborations on studies related to the molecular biology of cerebrovascular diseases. A standard FSGC operation manual was developed after a series of consensus conferences joining by an expert panel (5 stroke neurologists and 3 epidemiologists) and revised after a pilot study completed in three hospitals (2 medical centers and 1 regional hospital). All the research assistants and study nurses from the participating hospitals were trained on the standard procedure of case enrollment, including structured questionnaire and blood sample collection. The hospitals of FSGC have successfully established the Taiwan Stroke Registry (TSR), which provided a comprehensive insight into stroke management [7]. Ten hospitals joined the FSGC between 2005 to 2010 (Additional file 1, section 1) and 1732 stroke patients (84.3% ischemic, 5.5% transient ischemic attack[TIA] and 9.6% hemorrhagic) were recruited from FSGC hospitals and admitted to the Department of Neurology. Among 1460 ischemic stroke patients, there were 1103(75.5%) first-ever stroke and 357 (24.5) recurrent stroke. The criteria for the diagnosis of stroke have been detailed in previous study [7]. Ischemic stroke is an onset of focal neurological deficit with signs or symptoms persisted longer than 24 hours with or without acute ischemic lesion(s) on brain CT or with acute ischemic diffusion-weighted imaging lesion(s) on MRI that corresponded to the clinical presentations. TIA is defined as a transient focal neurologic deficit of ischemic causes that resolved within 24 hours. The subtypes of ischemic stroke were classified according to the Trial of Org 10172 in Acute Stroke Treatment (TOAST) criteria [8]. In the present case-control study, the subjects were composed of 765 first-ever ischemic stroke patients, who entered FSGC between 2005 and 2009. A total of 2736 healthy subjects were recruited as possible controls from a community-based prospective study of the nutrition health education program in Taipei City [9] and subjects who underwent physical examinations at TMUH during 2008-2009. Among these participants, 53 subjects with prevalent stroke were excluded. 977 age-matched controls were randomly selected in the remaining 2683 candidates. The study was approved by the ethics committees of the participating hospitals and Taipei Medical University on the understanding that all data would be coded and patient anonymity guaranteed. Informed consent was obtained from all participants and/or their relatives.

The design of the present study constitutes a two-step approach. In the primary analysis, we have conducted a case-control study with 765 Taiwanese patients who had first-ever ischemic stroke and 977 age-matched stroke-free controls for the two SNPs, rs12425791 and rs11833579 on chromosome 12p13. In the second step, the analysis on the association between these two SNPs and stroke-related mortality as well as stroke recurrent events were restricted only to ischemic stroke patients.

### Data collection

Data were collected from multiple centers as part of the TSR and applied to the FSGC for the improvement of sample quality and quantity [7]. FSGC-trained neurologists and study nurses collected preadmission data, inpatient elements, discharge information, and follow-up at 1, 3, 6, and 12 months, an English translation of a sample form found in Additional file 1, section 2. Investigators who were in charge of assigning a National Institutes of Health Stroke Scale (NIHSS) score had certified by the FSGC and trained to complete data entry through a web-based FSGC database system. In healthy controls, data on age, sex, and presence of major vascular risk factors were compiled by trained investigators using a structured questionnaire. Subsequently, the data were extracted from medical charts or self-reported during interview under trained research assistants/nurses. The definitions of risk factors were described in Additional file 1, section 3.

### Outcome data acquirement

Mortality rates were ascertained based on death-certificate data recorded at the Department of Health, Executive Yuan of Taiwan. Data of recurrent stroke were obtained in the history and examinations completed at 1, 3, 6, and 12 months follow-up in the FSGC data bank.

### Genotyping

Genomic DNA was extracted using the phenol/chloroform method and then stored at -80°C until use. SNPs were genotyped by Taqman fluorescent allelic discrimination assay with the ABI Prism 7900HT sequence detection system (Applied Biosystems, Foster City, CA, USA) Specific Taqman probes and primers were obtained from Applied Biosystems Assay-by-DesignTM service for SNP genotyping. The genotyping success rate was greater than 98% for all SNPs. To assure data quality, we sequenced 5% of samples using the ABI 3100 DNA sequencer (Applied Biosystems). The genotype concordance rate between duplicate samples was 100%.

### Statistical analysis

Student's t-test was used to compare continuous variables between cases and controls. The chi-square test was applied to test differences in categorical variables. Hardy-Weinberg equilibrium test for each SNP in healthy controls and cases were also assessed by chi-square analysis. To estimate the odds ratio (OR) and 95% confidence interval (CI), a multivariate logistic regression was applied under the adjustment of potential confounders. For each SNP, we tested for association under additive, co-dominant dominant and recessive models. The Cox proportional hazard model was used to assess the effect of individual SNPs on survival, the time of the stroke to the date of stroke-related death or recurrence-free survival time, the time from initial stroke occurrence to stroke recurrence. Hazard ratios (HRs) and 95% CI were estimated by fitting the Cox model while adjusting for stroke-related risk factors, including age, sex, hypertension, diabetes, cigarette smoking and alcohol drinking status, obesity and NIHSS score at beginning. Kaplan-Meier curves and log-rank tests were used to assess the differences in overall survival and recurrence-free survival by individual polymorphisms. We performed random-effect meta-analysis to pooled OR/HR and 95% CI across previous published studies which explored the association between SNPs on chromosome 12p13 and ischemic stroke [1-5]. All statistical analyses were performed with SAS Genetics software (version 9.1; SAS Institute, Cary, NC) or STATA [10]. Two-tailed p values were calculated and statistical significance was set at p-values < 0.05 nominally. To account for multiple comparison, we calculated the false discovery rate (FDR) using the Benjamini-Hochberg procedure and expressed using q-values [11].

## Results

Table 1 illustrates the basic characteristics of the study participants. The average ages of the ischemic stroke patients and healthy controls were 62.8 ± 12.2 and 63.4 ± 11.9 years, respectively. The distribution of gender among cases and controls were similar. There were a greater proportion of hypertensives, diabetics, smokers, alcohol users, and subjects with heart disease in the patient group compared with the controls. Mean BMI, fasting glucose levels, and LDLC were higher in cases than in controls while HDLC and cholesterol were lower in cases compared to controls. Among the 765 ischemic stroke events, median NIHSS was 3. The majority of them were due to small vessel occlusion (SVO, 44.7%). Other ischemic stroke types in order of frequency were large artery atherosclerosis (LAA, 26.8%), undetermined pathogenesis (14.0%), cardioembolism (CE, 7.1%), and strokes due to specific rare etiologies such as dissection, moyamoya syndrome etc. (2.6%). Genotype frequencies of SNP rs12425791 and rs11833579 were listed and in HWE with χ2 test p-values > 0.05. The allele frequencies of both SNPs were also shown in Table 1.

**Table 1:** Characteristics of patients with first-ever ischemic stroke and healthy control subjects

Characteristic	Cases (n = 765)	Controls (n = 977)	P-value
Age, mean (SD), y	62.8 ± 12.2	63.4 ± 11.9	0.526
Male, %	66.1	67.9	0.480
Hypertension, %	71.1	53.9	< 0.0001
Diabetes mellitus, %	43.2	16.1	< 0.0001
Heart disease, %	25.8	15.8	< 0.0001
Ever smoking, %	51.4	31.8	< 0.0001
Ever drinking, %	25.0	15.3	< 0.0001
Body mass index, mean (SD), kg/m^2^	25.3 ± 3.9	24.7 ± 3.3	0.001
Fasting glucose, means (SD), mmol/L	6.9 ± 2.7	5.8 ± 1.7	< 0.0001
HDL-C, mean (SD), mmol/L	1.1 ± 0.4	1.3 ± 0.3	< 0.0001
LDL-C, mean (SD), mmol/L	3.3 ± 1.0	3.2 ± 0.8	0.024
Triglyceride, mean (SD), mmol/L	1.6 ± 1.1	1.5 ± 1.1	0.097
Cholesterol, mean (SD), mmol/L	5.0 ± 1.2	5.2 ± 0.9	0.005
NIHSS at baseline, median (IQR)	3(1-5)		
TOAST, %			
Large artery atherosclerosis	26.8		
Small vessel occlusion	44.7		
Cardioembolism	7.1		
Specific pathogenesis	2.6		
Undetermined pathogenesis	14.0		
Missing	4.8		
rs12425791, n(%)			
GG	447(58.4)	557(57.4)	0.091
GA	268(35.0)	371(38.2)	
AA	50(6.5)	43(4.4)	
rs11833579, n(%)			
GG	338(44.3)	433(44.9)	0.431
GA	325(42.6)	425(44.0)	
AA	100(13.1)	107(11.1)	

HDL-C: high density lipoprotein cholesterol; LDL-C: low density lipoprotein cholesterol; NIHSS: National Institutes of Health Stroke Scale; TOAST: Trial of Org 10172 in Acute Stroke Treatment

**Figure 1:**
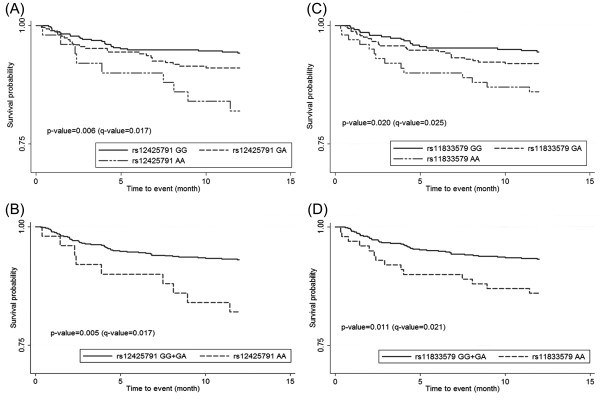
**Relationship between rs12425791 and rs11833579 and risk of death and/or recurrent stroke in additive and recessive models**. (a) rs12425791 under additive model. (b) rs12425791 under recessive model. (c) rs11833579 under additive model. (d) rs11833579 under recessive model.

Associations between SNPs and different types of ischemic stroke are shown in Table 2. After multiple testing corrections, there was no significant association between SNP rs12425791 and rs11833579 and ischemic stroke. However, the marginal significant association was observed between SNP rs12425791 and LAA under recessive model. Table 3 compares the distribution of rs12425791 and rs11833579 for patients with and without death and/or recurrent stroke during a 1-year follow-up period. Among 765 ischemic stroke patients, a total of 59 events (7.7%) were recorded, including 21 stroke-related deaths and 40 recurrent stroke events. There were 2 subjects who died among the recurrent stroke patients. Ischemic stroke patients carrying the rs12425791 GA (9.0%) and AA (18.0%) genotypes had higher event rates for death and/or stroke recurrence compared with participants with GG (5.8%) genotype (P < 0.05). Cox proportional model shown a significant association was observed for rs12425791 AA versus GG homozygote co-dominant model (HR, 3.30; 95%CI, 1.53-7.12; q-value, 0.018), for the dominant model (1.83; 95%CI, 1.08-3.08, q-value, 0.038), and for the recessive model (HR, 2.76; 95%CI, 1.34-5.68; q-value, 0.018). There is a similar tendency for the SNP rs11833579. In the sample of 59 events, 26 subjects are in the rs11833579 GA genotype group (8.0%) and 14 subjects are in the AA genotype group (14.0%). The prominently elevated risk was also found in rs11833579 AA versus GG homozygote co-dominant model (HR, 2.68; 95%CI, 1.31-5.48; q-value, 0.018) and the recessive model (HR, 2.15; 95%CI, 1.15-4.02, q-value, 0.034). The log-rank test shows a significant difference in event-free survival rates among patients with both SNPs during a 1-year follow-up period (Figure 1). The additional survival curve and log-rank test results were showed in Additional file 1, section 4.

**Table 2:** The odds ratios (ORs) of rs12425791 and rs11833579 genotypes for ischemic stroke

SNP	Total ischemic stroke				LAA			
	
	OR*	95% CI	P-value	q-value^†^	OR*	95% CI	P-value	q-value^†^
rs12425791								
Additive	1.00	(0.84-1.19)	0.977	0.977	1.03	(0.79-1.34)	0.831	0.997
Dominant	0.90	(0.72-1.11)	0.304	0.609	0.87	(0.63-1.19)	0.384	0.610
Recessive	1.67	(1.05-2.66)	0.031	0.183	2.30	(1.22-4.34)	0.010	0.062
								
rs11833579								
Additive	1.01	(0.87-1.18)	0.860	0.977	1.00	(0.79-1.26)	0.999	0.999
Dominant	0.95	(0.77-1.17)	0.596	0.893	0.88	(0.64-1.20)	0.407	0.610
Recessive	1.21	(0.88-1.67)	0.241	0.609	1.35	(0.85-2.14)	0.200	0.599

*: adjustment for age, gender, hypertension, diabetes mellitus, heart disease, dyslipidemia, cigarette smoking or alcohol drinking, and obesity

†: False discovery rate (FDR) was controlled using the Benjamini-Hochberg procedure

LAA, large artery atherosclerosis

**Table 3:** Survival analysis for rs12425791 and rs11833579 and risk of death or stroke recurrence among first-ever ischemic stroke patients

SNP	Neither death nor stroke recurrence observed (n = 706)		Either Death or stroke recurrence (n = 59)				
				
	**No**.	(%)	**No**.	(%)	HR* (95% CI)	P-value	q-value‡
**rs12425791**							
GG	421	(93.1)	26	(5.8)	1.0†		
GA	244	(90.7)	24	(9.0)	1.55(0.88-2.74)	0.131	0.149
AA	41	(82.0)	9	(18.0)	3.30(1.53-7.12)	0.002	0.018
GG	421	(94.2)	26	(5.8)	1.0		
GA+AA	285	(89.6)	33	(10.4)	1.83(1.08-3.08)	0.024	0.038
GG+GA	665	(93.0)	50	(7.0)	1.0		
AA	41	(82.0)	9	(18.0)	2.76(1.34-5.68)	0.006	0.018
**rs11833579**							
GG	319	(94.4)	19	(5.6)	1.0†		
GA	299	(92.0)	26	(8.0)	1.53(0.84-2.80)	0.164	0.164
AA	86	(86.0)	14	(14.0)	2.68(1.31-5.48)	0.007	0.018
GG	319	(94.4)	19	(5.6)	1.0		
GA+AA	385	(90.6)	40	(9.4)	1.80(1.03-3.13)	0.039	0.052
GG+GA	618	(93.2)	45	(6.8)	1.0		
AA	86	(86.0)	14	(14.0)	2.15(1.15-4.02)	0.017	0.034

*: Adjusted for age, gender, hypertension, diabetes mellitus, cigarette smoking and alcohol drinking, obesity, and NIHSS at the beginning

†: p for trend < 0.05

‡: False discovery rate (FDR) was controlled using the Benjamini-Hochberg procedure

## Discussion

The present study investigated the relationship between the SNP rs12425791 and rs11833579 on chromosome 12 and ischemic stroke and poor prognosis. The results showed there was a marginal significant association between SNP rs12425791 and LAA under recessive model. Previous studies concerning the relationship between SNP rs12425791 and rs11833579 and ischemic stroke were controversial. The GWAS finding and a large Japanese case control study presents an association between *NINJ2 *SNPs and ischemic stroke [1,5]. Although we observed that the SNP rs12425791 and rs11833579 on chromosome 12 were not significantly associated with ischemic stroke, the increased ischemic stroke risk trend were found among subjects carried the homozygous variant allele of these two SNPs. On the contrary, the results from a meta-analysis performed by ISGC and WTCCC2 including samples of European ancestry, a small young Italian case-control sample, and a recent report on 3 Swedish case-control samples did not find an association [2-4]. The possible reasons for the difference might be ethnicity (European Caucasian versus Asian) or study design (incident/first-ever ischemic stroke versus prevalent stroke). However, different ethnicity is unlikely the concern for different results since the chromosome 12p13 SNPs were significantly associated with ischemic stroke in both European Caucasians and Asian [1,5]. In addition, the MAF of SNP rs12425791 in our control subjects (23.5%) was similar with Han Chinese in Beijing, China (CHB) from the International HapMap project (24%), and the samples of European ancestry in the CHARGE (19%) [1], ISGC group (18-22%) [2] and the Swedish population (16-19%)[4]. For SNP rs11833579, the A allele frequency in the present study (32.9%) was 10% higher than in the CHARGE and ISGC group (23%)[1,2], but was similar to the CHB (30%).

Concerning to study design, we performed a meta-analysis combining results among all studies related to NINJ2 gene and ischemic stroke stratified by incident/first-ever ischemic stroke and prevalent stroke. The results found that a significant association between both SNPs on NINJ2 gene and ischemic stroke for (rs12425791, OR, 1.20; 95%CI, 1.04-1.38; for rs11833579, OR, 1.30; 95%CI, 1.10-1.54) among incident/first-ever ischemic stroke patients but not prevalent cases for (rs12425791, OR, 1.03; 95%CI, 0.99-1.07; for rs11833579, OR, 1.03; 95%CI, 0.99-1.08) in Additional file 1, section 5. Survival bias might be the reason for the lack of association between the SNPs and prevalent ischemic stroke. The result of meta-analysis might also correspond to our data which indicate that patients with the homozygous variant allele of these two SNPs had an increased risk of adverse outcomes following ischemic stroke. These two SNPs rs12425791 and rs11833579, located on chromosome 12p13, are in close proximity to the NINJ2 gene. Ninjurin 2 is a homophilic adhesion molecule expressed in many tissues including peripheral nerves, especially in Schwann cells and ganglion neurons [6,12]. It has been suggested that ninjurin 2, a nerve injury-induced protein, is involved in neuronal growth and play a role in nerve regeneration since it is up regulation during axotomy or nerve damage, promoting neurite outgrowth [6,13]. It is possible that the level of ninjurin 2 expression affects how the brain responds to cerebral ischemic insults. In an analogous manner, NINJ2 might be a candidate gene for stroke prognosis since it may influence the reaction to brain cells to injury. Our study found the initial NIHSS and mRS was not correlated with NINJ2 gene based upon genotype (data not shown), but further observed that first-ever ischemic stroke patients with the variant allele at these two SNPs had an increased risk of unfavorable outcome. Moreover, when we analyzed the relative change of modified Rankin Scale (mRS) during one year, we also found that 2.7% of subjects were regression (ΔmRS > 0) among patients with homozygous variant allele of SNP rs12425791 while only 0.7% of subjects with G allele became worse (data not shown). These evidence support the hypothesis of our study that the minor allele of NINJ2 gene might be related to greater stroke severity.

Several published studies following by the original research articles have encountered some limitations in sample size, multiple testing, population stratification, or publication bias [14,15]. In this study, a *post hoc *power calculation was done based on the survival probability were 10% and 5.6% among the variant allele carriers and the subjects with wild allele, respectively. The present sample size can reach to more than 80% power to detect both SNPs with the concluded genotypic HRs at the nominal Type I error rate less than 0.05. Also, we applied the false discovery rate method as multiple testing to prevent the false-positive association. Population stratification and publication bias may also lead to false-positive association. However, the limited evidence supports that population stratification and public bias is a minor issue for genetic association studies [16,17].

This study has several strengths. First, all ischemic stroke patients in our study had accurate diagnosis confirmed by CT/MRI. We also examined the association between SNPs and various subtypes of ischemic stroke based on the abundant ancillary information in the FSGC data bank. Moreover, the mean time interval between stroke onset and entry was remarkably short and completed in less than one day (mean+SD: 0.89 ± 1.70), thus reduced survivor bias, a significant problem in most stroke case series utilized for genetic analyses. Finally we could adjust our analyses for initial stroke severity since the NIHSS was administered in all cases prior to initiation of intravenous thrombolysis or other stroke therapies. However, there were some limitations in this study. Survival after stroke could depend on factors other than stroke severity, such as concomitant cardiac and respiratory disease. We have not examined the possible ranges of pleiotropic associations on the NINJ2 SNPs. In addition, the haplotype analysis of these two SNPs were not showed due to low linkage disequilibrium (r^2 ^= 0.53). According to the basis of the Hapmap data, the correlation coefficients were similar among Japanese populations (r^2 ^= 0.69). The identified SNP rs12425791 is more likely to be in linkage disequilibrium with the causal variants. Therefore, further detailed fine-mapping or re-sequencing in the region will be needed to identify the actual causal genetic variants.

## Conclusions

This study provides the first evidence that chromosome 12p13 are an independent predictor of mortality or stroke recurrence in patients with incident ischemic stroke in Taiwan. Further study is needed to explore the details of the physiological function and the molecular mechanisms underlying the association of this genetic locus with ischemic stroke.

## List of abbreviation

SNP: single nucleotide polymorphism; GWAS: genome-wide association study; CHARGE: Cohorts for Heart and Aging Research Consortium in Genetic Epidemiology; ISGC: International Stroke Genetics Consortium; WTCCC2: Wellcome Trust Case-Control Consortium 2; FSGC: Formosa Stroke Genetic Consortium; TSR: Taiwan Stroke Registry; TOAST: Trial of Org 10172 in Acute Stroke Treatment; NIHSS: National Institutes of Health Stroke Scale; OR: odds ratio; CI: confidence interval; HR: hazard ratio; FDR: false discovery rate; MAF: minor allele frequency; SVO: small vessel occlusion; LAA: large artery atherosclerosis; CE: cardioembolism; CHB: Han Chinese in Beijing; mRS: modified Rankin Scale

## Competing interests

The authors declare that they have no competing interests.

## Authors' contributions

HYC had full access to all of the data in the study and takes responsibility for the integrity of the data and the accuracy of the data analysis. HYC, YCH, SS and YHH were in charge of study concept and design. CHB, FIH, and CCY performed statistical analysis. CCY carried out the experiments. YCH was also responsible for drafting of the manuscript. HYC and SS have given final approval of the version to be published. WTC, HJL, HPT, LML, CJH, JSJ, SCT, and CIC devoted their time to the acquisition of data. All authors read and approved the final manuscript.

## Supplementary Material

Additional file 1 **additional files, figures and tables**. Click here for file
