# Supplementary material for: Association between genetic variant on chromosome 12p13 and stroke survival and recurrence: a one year prospective study in Taiwan
Source: J Biomed Sci. 2012 Jan 3;19(1):1. doi: 10.1186/1423-0127-19-1 (PMC3269363; doi:10.1186/1423-0127-19-1)
Supplement: Additional file 1 — additional files, figures and tables. [file 1423-0127-19-1-S1.DOC]

**Additional file 1**

1. **Section 1**

**List of FSGC Investigators:**

**National Taiwan University Hospital:** Jiann-Shing Jeng (Principal Investigator), Sung-Chun Tang, Shin-Joe Yeh, Li-Kai Tsai

**Shin Kong WHS Memorial Hospital:** Li-Ming Lien (Principal Investigator), Hou-Chang Chiu, Wei-Hung Chen, Chyi-Huey Bai, Tzu-Hsuan Huang, Chi-Ieong Lau, Ya-Ying Wu

**Taipei Medical University Hospital:** Rey-Yue Yuan (Principal Investigator), Chaur-Jong Hu, Jau- Jiuan Sheu, Jia-Ming Yu, Chun-Sum Ho

**Taipei Medical University** - **Wan Fang Hospital:** Chin-I Chen (Principal Investigator), Jia-Ying Sung, Hsing-Yu Weng, Yu-Hsuan Han, Chun-Ping Huang, Wen-Ting Chung

**Chi Mei Medical Center:** Der-Shin Ke (Principal Investigator), Huey-Juan Lin, Chia-Yu Chang, Poh-Shiow Yeh, Kao-Chang Lin, Tain-Junn Cheng, Chih-Ho Chou, Chun-Ming Yang

**Tri-Service General Hospital:** Giia-Sheun Peng (Principal Investigator), Jiann-Chyun Lin, Yaw-Don Hsu, Jong-Chyou Denq, Jiunn-Tay Lee, Chang-Hung Hsu, Chun-Chieh Lin, Che-Hung Yen, Chun-An Cheng, Yueh-Feng Sung, Yuan-Liang Chen, Ming-Tung Lien, Chung-Hsing Chou, Chia-Chen Liu, Fu-Chi Yang, Yi-Chung Wu, An-Chen Tso, Yu- Hua Lai, Chun-I Chiang, Chia-Kuang Tsai, Meng-Ta Liu, Ying-Che Lin, Yu-Chuan Hsu

**National Cheng Kung University Hospital:** Chih-Hung Chen (Principal Investigator), Pi-Shan Sung

**Taipei Veterans General Hospital:** Chang-Ming Chern (Principal Investigator), Han-Hwa Hu, Wen-Jang Wong, Yun-On Luk, Li-Chi Hsu, Chih-Ping Chung

**Lotung Poh Ai Hospital**: Hung-Pin Tseng (Principal Investigator), Chin-Hsiung Liu, Chun-Liang Lin, Hung-Chih Lin

**Taipei Medical University** - **Shuang Ho Hospital:** Chaur-Jong Hu (Principal Investigator)

1. **Section 2**

Table S1 Post stroke follow-up records

| □1 month □3 month □6 month □12 month post stroke follow up  Follow-up date: / /   - □lost to follow-up - Medical record I   □Death  □stroke causes (□cerebral infarction □cerebral hemorrhage)  □heart disease causes  □others   - Medical record II   □None  □recurrence of stroke  □cerebral infarction □cerebral hemorrhage)  Date: / / Hospital:  □heart disease  □Acute myocardial infarction □Angina □heart failure  □Others  Date: / / Hospital:   - Modified Rankin Scale: |
| --- |

1. **Section 3**

Measurement and definition of risk factors

Fasting serum total cholesterol, triglyceride, high-density lipoprotein cholesterol (HDLC), glucose, and uric acid concentrations were measured by an automatic analyzer (UniCel DXC 800, BeckMan). Low-density lipoprotein cholesterol (LDLC) was calculated using the Friedewald formula.^1^

1. Hypertension: either a systolic blood pressure ≥140 mmHg or diastolic blood pressure ≥90 mmHg, a previous diagnosis of HTN by a physician or the use of antihypertensive drugs.
2. Type 2 diabetes mellitus: either a fasting glucose value ≥126 mg/dL or a history of DM with current pharmacological treatment.
3. Dyslipidemia: either a positive history of dyslipidemia or anti-lipid drug use before recruitment or on an abnormal value for one of the following serum lipid measurements (cholesterol level ≥200 mg/dL, triglyceride level ≥150 mg/dL, LDLC ≥130 mg/dL or HDLC <40 mg/dL).
4. Obesity: a BMI ≥25 kg/m2 was used to define obesity.^2^
5. Cigarette Smoking: ever smoking versus never smoking.
6. Alcohol use: ever versus never.
7. **Section 4**

Figure S1. Relationship between (a)rs12425791 and (b)rs11833579 and risk of death and/or recurrent stroke in dominant models.

(a)


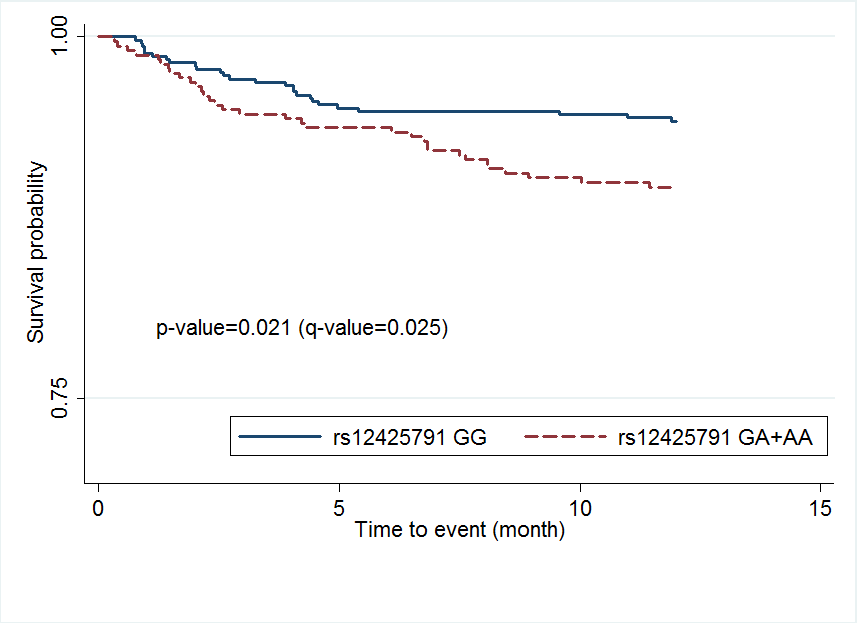


(b)


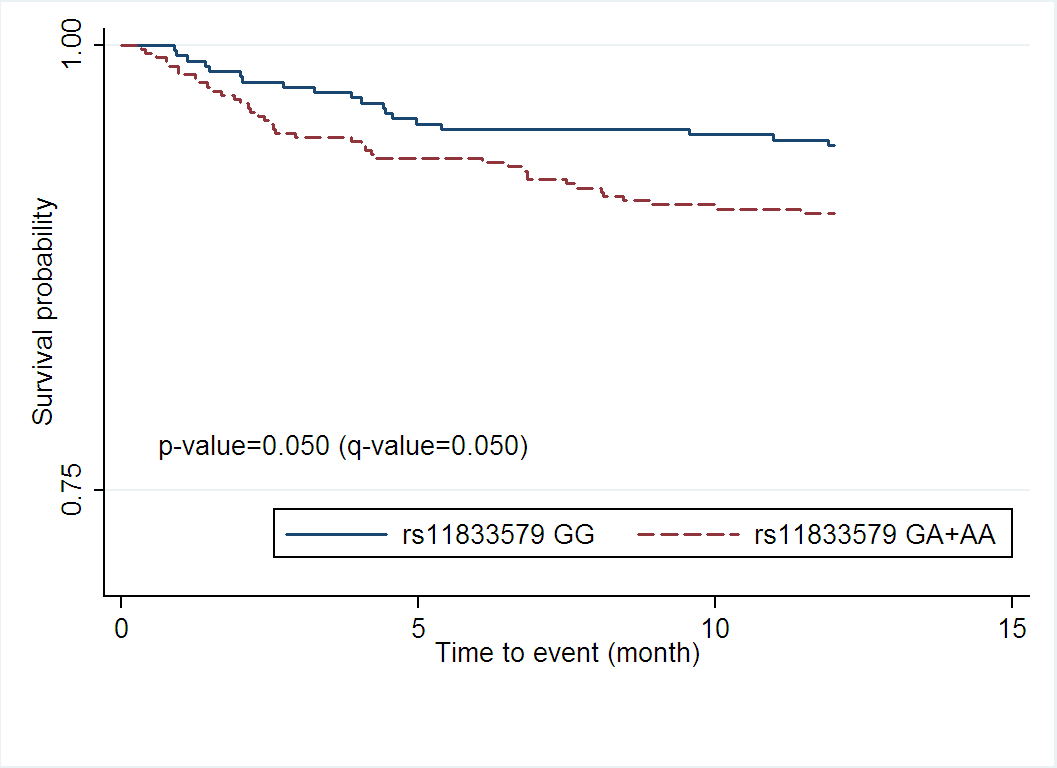


1. **Section 5**

Table S2 Studies on SNP rs12425791 in the Meta-analyses

| Source | Country or Study | Ethnicity | Stroke type | Minor Allele | Major allele | MAF |
| --- | --- | --- | --- | --- | --- | --- |
| Rosand et al., 2010^3^ | WGHS | European | Incident | A | G | 0.18-0.22 |
| IKram et al., 2009^4^ | ARIC | European | Incident | A | G | 0.19 |
| IKram et al., 2009^4^ | CHS | European | Incident | A | G | 0.19 |
| IKram et al., 2009^4^ | FHS | European | Incident | A | G | 0.19 |
| IKram et al., 2009^4^ | Rotterdam | European | Incident | A | G | 0.19 |
| Hsieh et al. | Taiwan (FSGC) | Asian | First-ever | A | G | 0.24 |
| Olsson et al., 2011^5^ | Sweden (LSR) | European | First-ever | A | G | 0.17 |
| Olsson et al., 2011^5^ | Sweden (MDC) | European | Incident | A | G | 0.16 |
| Matsushita et al., 2010^6^ | Japan | Asian | Prevalent | A | G | 0.34 |
| Rosand et al., 2010^3^ | China (SHINING) | Asian | Prevalent | A | G | 0.27 |
| Lotta et al., 2010^7^ | Italy | European | Prevalent | A | G | 0.24 |
| Olsson et al., 2011^5^ | Sweden (SAHLSIS) | European | Prevalent | A | G | 0.17 |
| Rosand et al., 2010^3^ | ISGC | European | Prevalent | A | G | 0.19 |
| Ding et al., 2011^8^ | China (Wuhan) | Asian | Prevalent | A | G | 0.24 |
| Tong et al., 2011^9^ | China (Shenzhen and Hailongjiang) | Asian | Prevalent | A | G | 0.23 |

WGHS, Women's Genome Health Study ; ARIC, Atherosclerosis Risk in Communities; CHS, Cardiovascular Health Study; FHS, Framingham Heart Study; FSGC, Formosa Stroke Genetic Consortium; LSR indicates Lund Stroke Register; MDC, the Malmö Diet and Cancer study; SHINING, Stroke Hypertension Investigation in Genetics; SAHLSIS, the Sahlgrenska Academy Study on Ischemic Stroke; ISGC, International Stroke Genetic Consortium

Table S3 Studies on SNP rs11833579 in the Meta-analyses

| Source | Country or Study | Ethnicity | Stroke type | Minor Allele | Major allele | MAF |
| --- | --- | --- | --- | --- | --- | --- |
| Rosand et al., 2010^3^ | WGHS | European | Incident | A | G | 0.23 |
| IKram et al., 2009^4^ | ARIC | European | Incident | A | G | 0.23 |
| IKram et al., 2009^4^ | CHS | European | Incident | A | G | 0.23 |
| IKram et al., 2009^4^ | FHS | European | Incident | A | G | 0.23 |
| IKram et al., 2009^4^ | Rotterdam | European | Incident | A | G | 0.23 |
| Hsieh et al. | Taiwan (FSGC) | Asian | First-ever | A | G | 0.33 |
| Matsushita et al., 2010^6^ | Japan | Asian | Prevalent | A | G | 0.42 |
| Lotta et al., 2010^7^ | Italy | European | Prevalent | A | G | 0.30 |
| Rosand et al., 2010^3^ | ISGC | European | Prevalent | A | G | 0.23-0.27 |
| Ding et al., 2011^8^ | China (Wuhan) | Asian | Prevalent | A | G | 0.33 |
| Tong et al., 2011^9^ | China (Shenzhen and Hailongjiang) | Asian | Prevalent | A | G | 0.33 |

WGHS, Women's Genome Health Study ; ARIC, Atherosclerosis Risk in Communities; CHS, Cardiovascular Health Study; FHS, Framingham Heart Study; FSGC, Formosa Stroke Genetic Consortium; ISGC, International Stroke Genetic Consortium

Table S4 Meta-analysis of SNP rs12425791 with incident/ first-ever ischemic stroke and prevalent ischemic stroke

| Stroke type | Odds Ratios | 95% CI | Z value | P-value | Heterogeneity p-value |
| --- | --- | --- | --- | --- | --- |
| **rs12425791** |  |  |  |  |  |
| Incident/ first-ever stroke | 1.196 | 1.037-1.381 | 2.455 | 0.014 | 0.999 |
| Prevalent stroke | 1.027 | 0.986-1.069 | 1.263 | 0.206 | 0.806 |
| **rs11833579** |  |  |  |  |  |
| Incident/ first-ever stroke | 1.297 | 1.096-1.536 | 3.018 | 0.003 | 0.640 |
| Prevalent stroke | 1.034 | 0.991-1.078 | 1.544 | 0.123 | 0.852 |

Figure S2. Forest plots showing association between SNP (a)rs12425791 and (b)rs11833579 and ischemic stroke in incident/ first-ever stroke

(a)


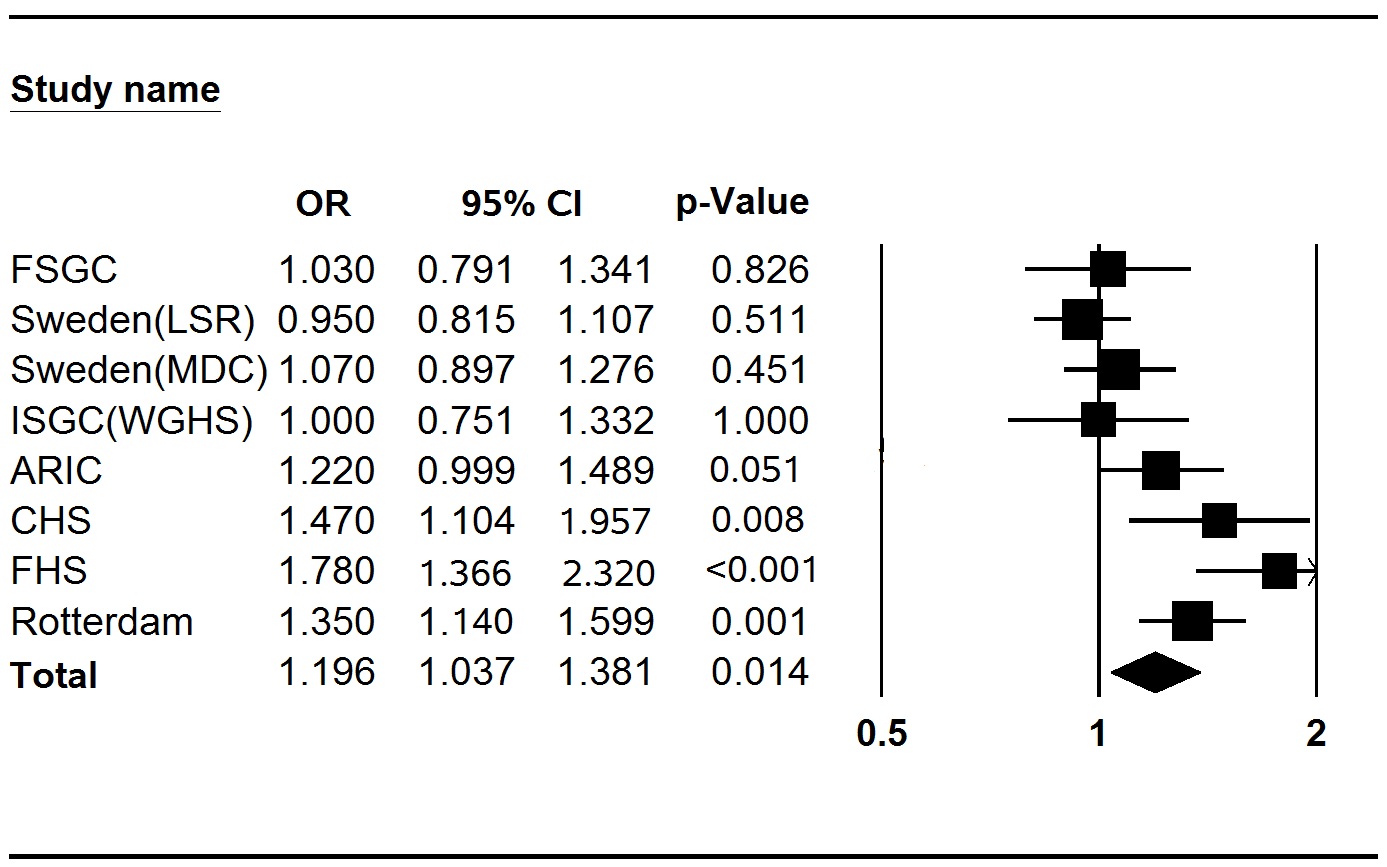


(b)


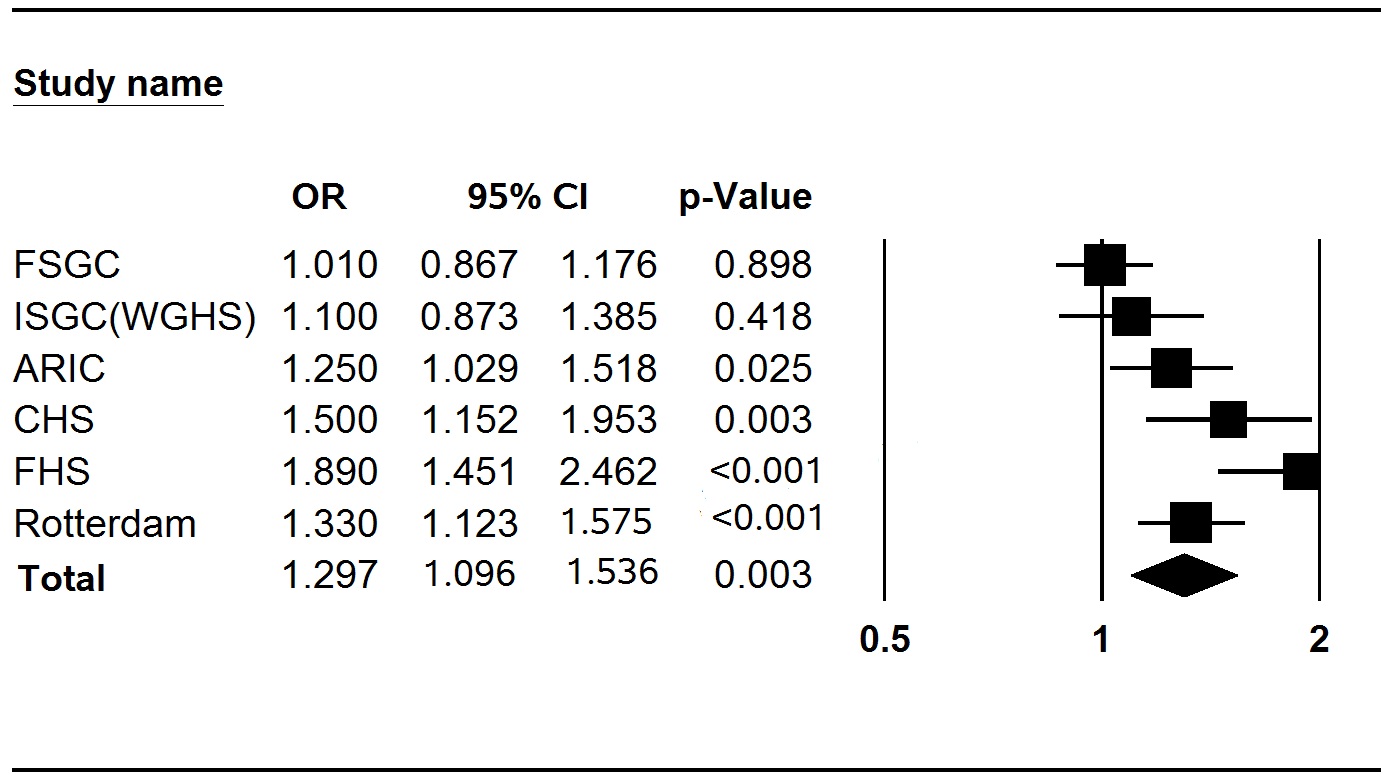


FSGC, Formosa Stroke Genetic Consortium; LSR, Lund Stroke Register; MDC, the Malmö Diet and Cancer study; ISGC, International Stroke Genetic Consortium; WGHS, Women's Genome Health Study ; ARIC, Atherosclerosis Risk in Communities; CHS, Cardiovascular Health Study; FHS, Framingham Heart Study

Figure S3. Forest plots showing association between SNP (a)rs12425791 and (b)rs11833579 and ischemic stroke in prevalent stroke

(a)


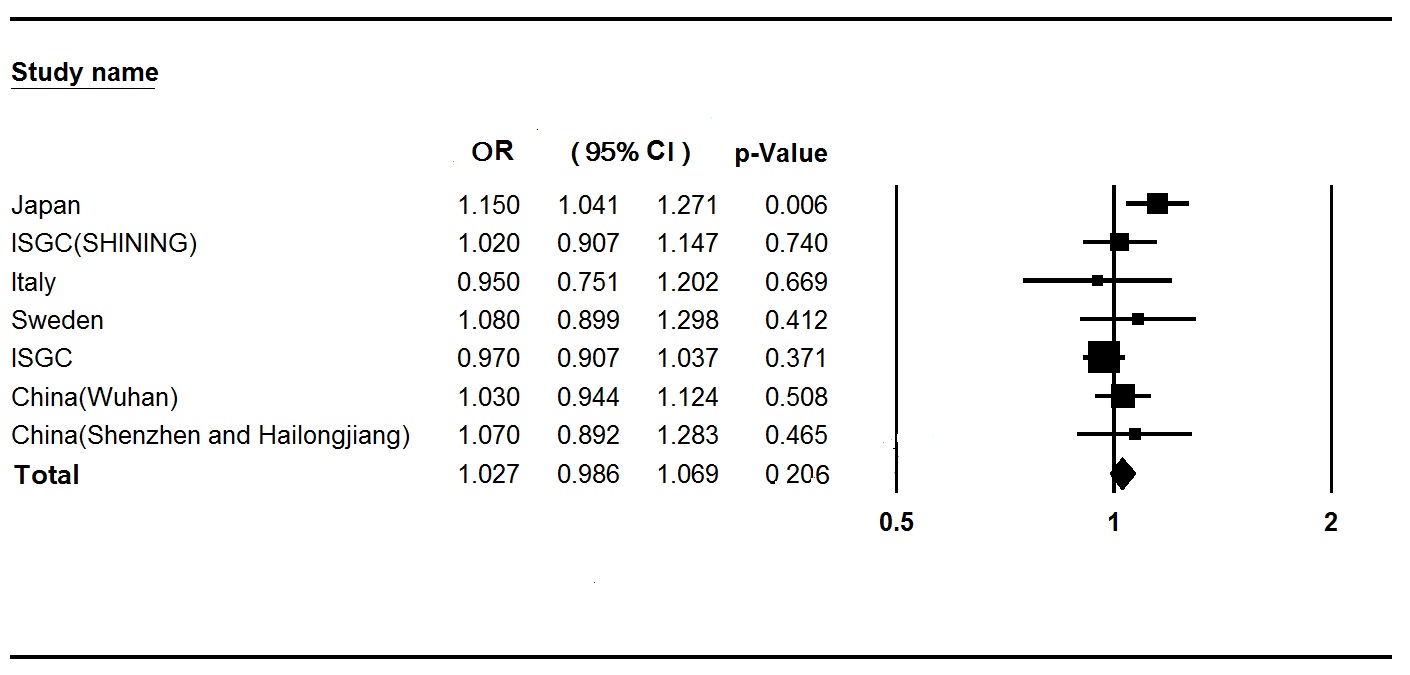


(b)


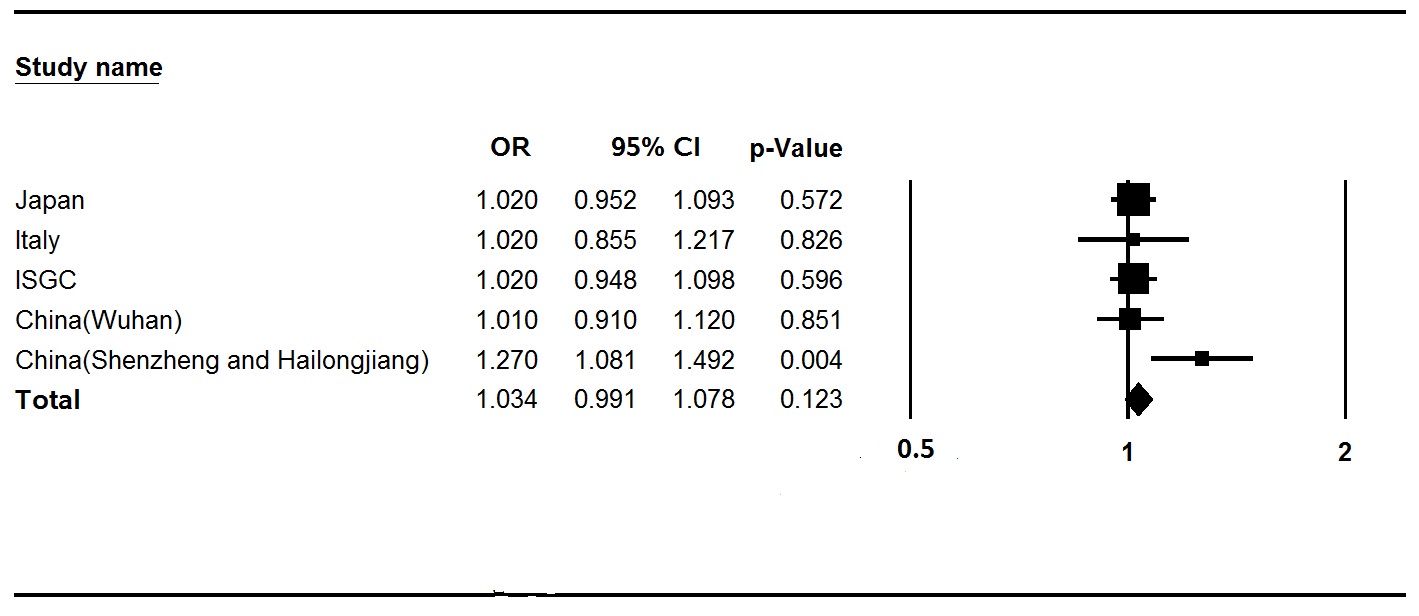


ISGC, International Stroke Genetic Consortium; SHINING, Stroke Hypertension Investigation in Genetics

Reference

1. Friedewald WT, Levy RI, Fredrickson DS: **Estimation of the concentration of low-density lipoprotein cholesterol in plasma, without use of the preparative ultracentrifuge.** *Clin Chem* 1972;**18**:499-502.
2. Wen CP, David Cheng TY, Tsai SP, Chan HT, Hsu HL, Hsu CC, Eriksen MP: **Are Asians at greater mortality risks for being overweight than Caucasians?** **Redefining obesity for Asians.** *Public Health Nutr* 2009;**12**:497-506.
3. International Stroke Genetics Consortium; Wellcome Trust Case-Control Consortium 2: **Failure to validate association between 12p13 variants and ischemic stroke.** *N Engl J Med* 2010;**362**:1547–1550.
4. Ikram MA, Seshadri S, Bis JC, Fornage M, DeStefano AL, Aulchenko YS, Debette S, Lumley T, Folsom AR, van den Herik EG, Bos MJ, Beiser A, Cushman M, Launer LJ, Shahar E, Struchalin M, Du Y, Glazer NL, Rosamond WD, Rivadeneira F, Kelly-Hayes M, Lopez OL, Coresh J, Hofman A, DeCarli C, Heckbert SR, Koudstaal PJ, Yang Q, Smith NL, Kase CS, Rice K, Haritunians T, Roks G, de Kort PLM, Taylor KD, de Lau LM, Oostra BA, Uitterlinden AG, Rotter JI, Boerwinkle E, Psaty BM, Mosley TH, van Duijn CM, Breteler MMB, Longstreth WT, Jr., Wolf PA: **Genomewide association studies of stroke.** *N Engl J Med* 2009;**360**:1718-1728.
5. Olsson S, Melander O, Jood K, Smith JG, Lövkvist H, Sjögren M, Engström G, Norrving B, Lindgren A, Jern C; International Stroke Genetics Consortium (ISGC): **Genetic variant on chromosome 12p13 does not show association to ischemic stroke in 3 Swedish case-control studies.** *Stroke* 2010;**42**:214-216.
6. Matsushita T, Umeno J, Hirakawa Y, Yonemoto K, Ashikawa K, Amitani H, Ninomiya T, Hata J, Doi Y, Kitazono T, Iida M, Nakamura Y, Kiyohara Y, Kubo M: **Association study of the polymorphisms on chromosome 12p13 with atherothrombotic stroke in the Japanese population.** *J Hum Genet* 2010;**55**:473–476.
7. Lotta LA, Giusti B, Saracini C, Vestrini A, Volpe M, Rubattu S, Peyvandi F: **No association between chromosome 12p13 single nucleotide polymorphisms and early-onset ischemic stroke.** *J Thromb Haemost* 2010;**8**:1858–1860.
8. Ding H, Tu X, Xu Y, Xu C, Wang X, Cui G, Bao X, Hui R, Wang QK, Wang DW: **No evidence for association of 12p13 SNPs rs11833579 and rs12425791 within NINJ2 gene with ischemic stroke in Chinese Han population.** *Atherosclerosis* 2011;**216**:381-382.
9. Tong Y, Zhang Y, Zhang R, Geng Y, Lin L, Wang Z, Liu J, Li X, Cao Z, Xu J, Chai Y, Fan H, Hu FB, Lu Z, Cheng J: **Association between two key SNPs on chromosome 12p13 and ischemic stroke in Chinese Han population.** *Pharmacogenet Genomics* 2011;**21**:572-578.
